# Telomeres in mitosis: organization and dynamics

**DOI:** 10.1080/19491034.2026.2698214

**Published:** 2026-09-18

**Authors:** Mattia La Torre, Serena Fabiano, Romina Burla, Isabella Saggio

**Affiliations:** a Department Biology and Biotechnology Charles Darwin Sapienza, University of Rome, Rome, Italy; b Institute of Molecular Biology and Pathology, CNR, Rome, Italy; c Istituto Pasteur Fondazione Cenci Bolognetti, Rome, Italy

**Keywords:** ESCRT, genomic instability, mitosis, nuclear organization, telomeres

## Abstract

Telomeres are specialized chromosomal structures that protect chromosome ends and maintain genome stability. Their organization depends on telomeric DNA repeats, the shelterin complex, accessory factors, and telomerase. While telomere structure and maintenance have been characterized, their dynamics during mammalian mitosis remain incompletely understood. Mitosis involves profound cellular reorganization, including chromatin condensation, spindle formation, and nuclear membrane disassembly and reassembly. Emerging evidence suggests that telomeres participate in these processes and may contribute to proper chromosome segregation and post-mitotic chromatin organization. Studies indicate that telomeric proteins such as TRF1 and TNKS1 interact with centrosomes and spindle components, while telomeres also interface with chromatin and nuclear membrane reassembly factors, including BAF1, LAP2α, and the ESCRT machinery during late mitosis. Defects in these processes or interactions can lead to telomere dysfunction and genomic instability. Understanding telomere dynamics during mitosis is therefore critical for elucidating mechanisms underlying aging, laminopathies, and other telomere-related human diseases.

## Organization of telomeres

Back in the 1930s, experiments in fly and in maize suggested that chromosome ends were protected to avoid aberrations and fusions [1,2]. Later studies in yeast showed the presence of tandem repeats at chromosome ends [3,4]. In the 1980s, the Nobel prize winners Blackburn and Greider integrated the functional picture of telomeres by identifying the enzyme elongating chromosome ends, the telomerase [5]. In the last fifty years, a large body of evidence has pictured the organization of chromosome ends, established a list of telomere-associated factors, and defined the evolutionary conserved mechanisms involved in telomere maintenance versus the non-conserved ones.

**Figure 1.**
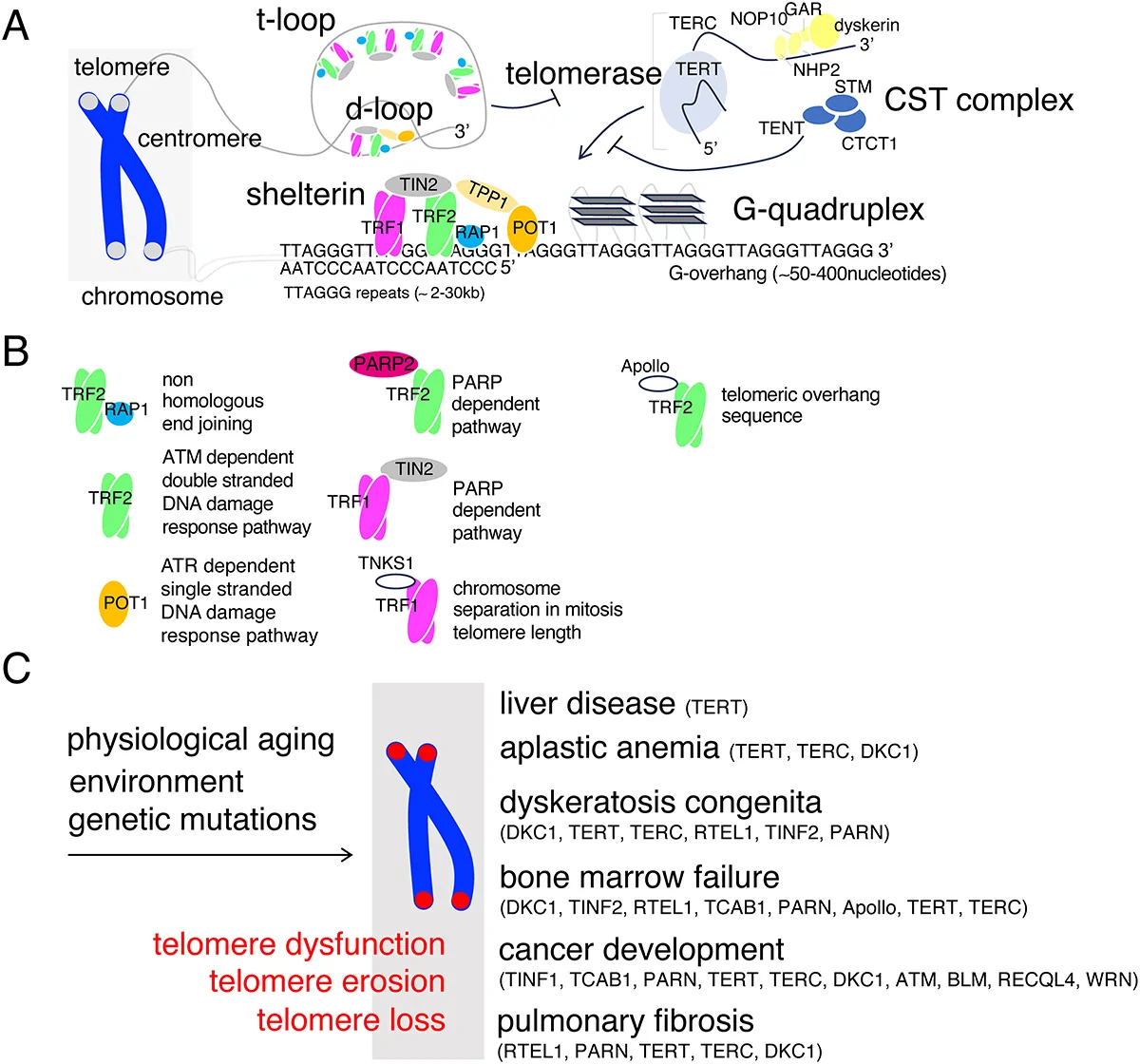
Telomere organization and association with disease.

A telomeric prototypical characteristic, found from yeast to mice and from fly to humans, is that telomeric DNA includes tandem repeats. Indeed, human telomeric DNA is composed of double-stranded repeats of the hexamer TTAGGG, followed by a 50–400 nucleotide long, G-rich region of single-stranded DNA [6–9]. This peculiar nature of telomeric DNA suggested its arrangement into a T-loop structure [10–12]. Data also indicate that the telomeric G-enriched DNA forms G quadruplex structures (Figure 1(A)) [13].

(A-B) Schematic representation of telomere organization (A) and the contribution of single telomeric-associated components to molecular processes (B). (C) Physiological aging, environmental stress, and genetic mutations lead to telomere erosion, dysfunction, and loss, which are associated with a spectrum of human diseases, including liver disease, aplastic anemia, dyskeratosis congenita, bone marrow failure, cancer, and pulmonary fibrosis.

Besides telomeric and subtelomeric DNA sequences themselves, many proteins contribute to telomere organization and function. The best characterized among these, are six factors named shelterins. Human shelterins are: TRF1, TRF2, POT1, Rap1, TIN2 and TPP1. These form a complex in somatic cells in which TRF1 and TRF2 bind to TIN2, TIN2 interacts with TPP1, which also binds to POT1. TRF1 and TRF2, in turn, bind to double-stranded telomeric DNA, while POT1 binds to single-stranded telomeric DNA [14–22]. Rap1 is connected to the shelterin complex by binding TRF2 (Figure 1(A)) [23]. Although the precise stoichiometry of the shelterin complex is not yet defined, it has been established that TPP1 and POT1, in humans, are significantly lower in copy number as compared to the other members of the complex [24]. The shelterin complex controls telomere metabolism and protects telomeres. For this latter aspect, the six factors shelter chromosome ends from the operativity of the DNA damage response pathways, that can be detrimental for telomere metabolism. In other words, shelterins protect chromosome ends from the fact that, in the absence of accurate protection, they would be recognized as damaged DNA sites. Rap1 and TRF2 protect telomeres from non-homologous end joining [25,26]. TRF2 acts on the ATM-dependent double-stranded DNA damage response pathway, while POT1 acts on the ATR-dependent single-stranded DNA damage response pathway [27–37]. Finally, TRF2 and TIN2, protect telomeres from the PARP-dependent pathway (Figure 1(B)) [38,39], depending, respectively, on direct interaction [40], or via TRF1 [12].

Beyond shelterins, telomere metabolism requires multiple accessory factors and the telomerase. A pivotal shelterin accessory factor is TNKS1, a TRF1-binding protein that regulates chromosome separation in mitosis and telomere length [41–44]. Interestingly, TNKS1 is present in human but not in mouse cells. Another accessory complex playing an important role in telomere metabolism is the CST. This is composed of the Cdc13, Stn1 and Ten1 factors, and was first identified in yeast (Figure 1(A)) [45,46]. Its role is played in coordination with shelterins and contributes to regulate telomeric replication [47–50]. Finally, another key accessory telomeric factor is Apollo, a nuclease interacting with TRF2 and controlling the telomeric overhang sequence (Figure 1(B)) [51–53].

Telomerase is the holoenzyme complex that mediates the elongation of telomeres. Human TERT -telomerase reverse transcriptase- corresponds to the catalytic component of the complex, while TERC -telomerase RNA component- serves as template for the enzymatic activity (Figure 1(A)) [54–57]. The enzyme is highly expressed in embryonic stem cells and moderately in some adult (stem) cells [58–61]. The interest into the understanding of the properties of telomerase evidently intercepts the concept of telomere length and cell senescence and is largely reported in the literature.

In humans, telomeres have been associated with multiple diseases. Physiological aging, environment, or genetic mutations can drive telomere erosion, telomere dysfunction or loss. Dysfunctional telomeres have been associated with bone marrow failure, dyskeratosis congenita [62–64], aplastic anemia [65], pulmonary fibrosis [66,67], liver disease [68] and, in broader terms, with cancer (Figure 1(C)) [69–75]. Given these associations, it is not surprising that, since the identification of telomeres and telomerase, a large body of data is being continuously generated both to deepen the understanding of the mechanisms involved in telomere maintenance and to define those affecting their function. However, while studies on the length of telomeres are abundant, along with those on telomere structural organization and maintenance mechanisms, the dynamics of telomeres, especially during mitotic division of somatic human cells, is yet incompletely elucidated.

## Dynamic elements in mitotic division

Mitosis in eukaryotic cell division is preceded by the G1, S and G2 phases. The G1 prepares cells to DNA replication, which happens in the S (synthesis) phase. During the S phase chromosomes and centrosomes are duplicated. At this stage, the cohesin and the condensin complexes locate on chromosomes to help the subsequent mitotic phase right after the G2 [76–78]. The mitotic phase is a central and complex task for the cell (and for the organism). Indeed, for example, during the early stages of mitosis the long DNA molecule must be reorganized in its size of about 100,000-fold and the duplicated strands must be carefully subdivided in the two daughter cells and nuclei. If this does not happen correctly a reduced and/or excessive or altered DNA content is inherited by the daughter cells [79]. These conditions have been associated with numerous diseases, of which the most extensively studied is cancer [78,80,81].

Successful distribution of the post replication compacted, and duplicated DNA content is guaranteed by multiple mechanisms and structures, starting in prophase. A key role is played by the spindle microtubules and by the connected motors. The radius of movement that must be covered by the chromosome/chromatids along with the accuracy required for their distribution to the daughter cells are especially challenging activities [82–84]. Further, the spindle microtubules regulate the distribution into the daughter cells of organelles including mitochondria, the Golgi apparatus, and centrosomes [85,86]. On top of this, in open mitosis -from now on mitosis- the nuclear membrane needs to be disassembled and successively reassembled to reach the post-mitotic interphase reorganization. These events are intimately intermingled with DNA structural organization and must be considered altogether [79]. These facts taken together anticipate the complexity of the mechanisms and factors involved in mitosis.

Numerous studies have explored the different mechanistic angles and molecular mediators of the mitotic process. However, the picture is not yet complete. Some questions are still under investigation. One of these concerns the clarification of telomere dynamics during mitosis, their molecular links with the different mitotic players, along with the role of telomeres in determining post-mitotic chromatin reorganization.

## Early stages of mitotic division

In the first phase of mitosis, the prophase, chromatin condensation decreases the length of DNA and chromosome/chromatids become cytologically recognizable. Chromatin condensation is associated with post translational modifications and epigenetic mark variations that alter the chemical properties of the DNA and allow its super-organization around histone octamers. Condensins contribute to this latter process by further affecting DNA folding [87–90].

**Figure 2.**
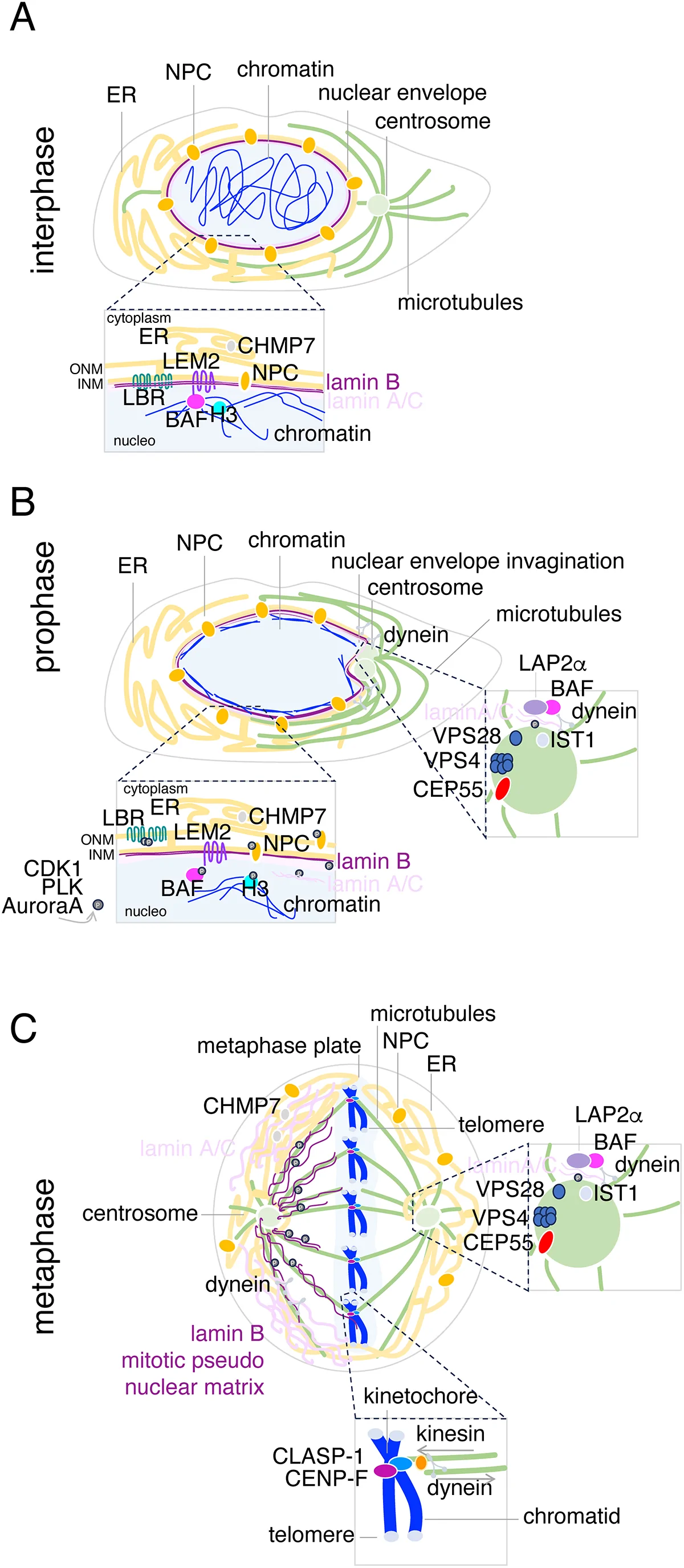
Early stages of mitotic division. (A-C) Schematic representation of the structural organization of a mammalian cell during interphase (A), prophase (B) and metaphase (C). During prophase, cell components undergo major changes. The nuclear envelope disassembles, DNA condenses, and membrane elements change their organization and/or location in response to phosphorylation events (A-B, insets). The microtubules-associated motors (dynein, kinesin) exert forces to dismantle the nuclear membrane. Several molecular factors localize and contribute to the organization of centrosomes and of the spindle during mitosis (B-C, insets). During metaphase, chromosomes/chromatids congress at the metaphase plane and are attached to spindle microtubules via kinetochores (C, inset). Lamin B persists as a mitotic pseudo nuclear matrix, while ESCRT and other factors localize to spindle (C, inset). NPC, nuclear pore complex; ER, endoplasmic reticulum; ONM, outer nuclear membrane; INM, inner nuclear membrane.

Beyond that of DNA, in mammalian cells, another major cytological transformation occurs during prophase, the disassembly of the nuclear membrane. This structure cages and protects the DNA in interphase and is characterized by an inner and outer nuclear membrane (Figure 2(A)). In addition, the nuclear membrane hosts membrane associated proteins - synthesized at the endoplasmic reticulum - and the multiprotein nuclear pore complexes. Beneath the nuclear membrane resides the lamina, which is in close contact with chromatin (Figure 2(A)) [91,92]. Mainly via phosphorylation, during prophase, these membrane elements change their organization and/or location (Figure 2(B)) [93]. For example, the membrane-associated factor LBR and the nuclear pore complex components POM121, NDC1 and NUP201, become embedded in the endoplasmic reticulum [94–98]. On the other hand, B-type lamins, which are anchored in the membranes with a farnesyl group, remain associated and create a mitotic pseudo-nuclear membrane [99]. Phosphorylation operates as well for the mobility of chromatin-associated factors including the BAF1 and histone H3 [100,101]. The phosphorylation events occurring during prophase are regulated by several protein kinases as CDK1, PLK and Aurora A (Figure 2(B)) [102–106]. These kinases are also regulators of the assembly of the γ tubulin ring, a structure composing the microtubule organizing center, driving microtubule spindle assembly [107,108]. At the same time, also via a phosphorylation regulatory process, the microtubule cytoplasmic network dissolves [109–111]. While cytoplasm microtubules disappear and new microtubules form to compose the spindle, cytokeratins remain and surround the nucleoplasm [112]. Finally, during prophase the Golgi complex is fragmented, and the Golgi-secretory mechanisms are suspended [113–116]. Centrosomes at this stage are both separated or still close one to the other. Independently from this latter characteristic, once the nuclear membrane is not cytologically present the prophase is considered concluded (Figure 2(B)) [79,117].

During metaphase, chromosomes/chromatids congress at the metaphase plane (Figure 2(C)). The spindle drives proper attachment and consequent distribution of chromatids in the daughter cells [118,119]. The organization of the spindle is dependent on the duplication of centrosomes and on their distribution at opposite poles [120]. The first element favoring the correct distribution is that microtubules of the spindle are organized from the two spindle poles, therefore from opposite directions. The dynamic instability of microtubules is the second key characteristic mediating their function. Indeed, microtubules can rupture, grow again or shorten [121–125]. Moreover, they can rotate around the spindle poles. Altogether microtubules operate a ‘search and capture’ activity on chromosomes/chromatids to dispose them in the most appropriate way for their distribution [121,126,127]. The attachment of chromosomes/chromatids to spindle fibers is based on kinetochores These are composed of a large range of factors assembling on specific chromatin portions. Factors include microtubule-binding proteins as CLASP1 and CENP-F, and molecular motors with minus and plus end directions, as dyneins and kinesins (Figure 2(C)) [128–132]. During metaphase, microtubules contribute also to the clearance of the spindle area, by, for example, removing the endoplasmic reticulum elements. The continuity between the endoplasmic reticulum and the nuclear membrane presumably facilitates this process, as its reversal at the end of mitosis, when the nuclear membrane needs to be recomposed [133–135]. A variety of factors is implicated in this membrane reorganization. Studies on the Endosomal Sorting Factor Complex (ESCRT) have suggested a role for some of its components into the organization of centrosomes and of the spindle during mitosis. Indeed, multiple ESCRT proteins localize to centrosomes, microtubule organizing centers, and/or to the mitotic spindle (Figure 2(C)) [136]. Moreover, in several cell models, the depletion of ESCRT subunits generates mitotic defects and multipolar spindles [136–138].

## Late stages of mitotic division

**Figure 3.**
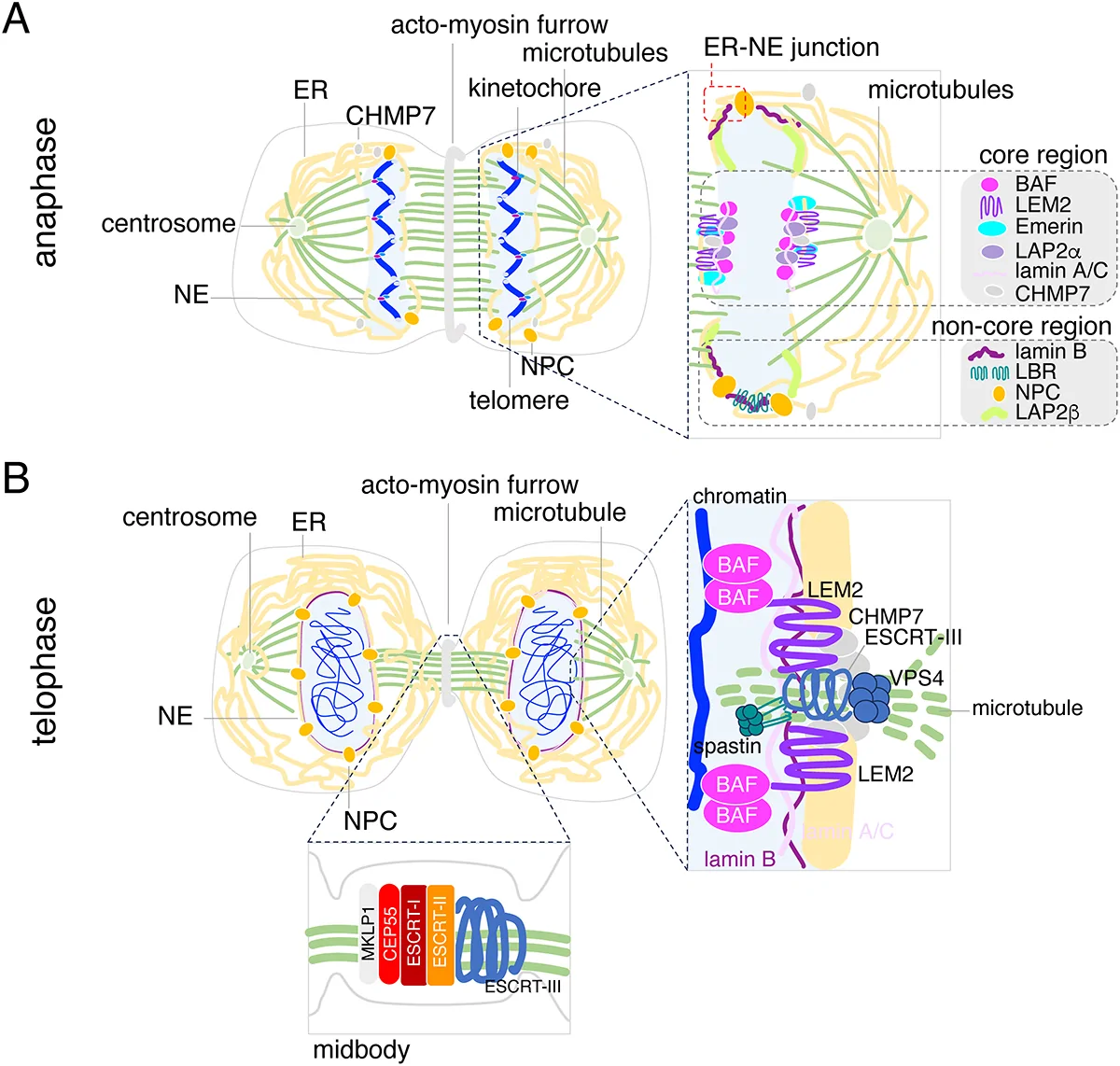
Late stages of mitotic division. (A-B) Schematic representation of a mammalian cell during anaphase (A) and telophase (B). During anaphase, sister chromatids segregate toward opposite poles through microtubule dynamics and motor activity, while the central spindle and midbody form and an acto-myosin furrow initiates cytokinesis. In parallel, nuclear envelope reassembly begins around chromosome sets, with recruitment of membrane components and nuclear pore complexes. Distinct core (BAF1, LEM, LAP2α, emerin, lamin A/C, CHMP7) and non-core (NPC, lamin B, LBR, LAP2β) specific proteins are recruited to initiate the reassembly of the nuclear envelope. During telophase, ESCRT machinery (via LEM2-CHMP7) promotes nuclear envelope sealing and spindle disassembly through spastin recruitment (B, inset), while ESCRT structures at the midbody drive abscission and final cell separation (B, inset). ER, endoplasmic reticulum; NE, nuclear envelope; NPC, nuclear pore complex.

The metaphase is considered concluded when sister chromosome/chromatids start to separate and the entry into anaphase is allowed only if chromosomes/chromatids have initiated their distribution into the daughter cells. This is controlled by multiple factors, including securin, cyclin B and CDK1 [139–146]. Shortening of microtubules and the activity of molecular motors drive the movement of chromatids -now chromosomes- to the two poles [147–149]. On the other hand, microtubule fibers increase in numbers in the area between the two chromosome sets, and the midbody, the structure bridging the two daughter cells prior to cytokinesis, starts to become detectable. An acto-myosin furrow forms in late anaphase at this site, creating a progressively thinner bridge that is subsequently cut to free the two daughter cells one from the other (Figure 3(A)) [150–157].

It is in anaphase that the nuclear membrane starts its reorganization around the two chromosome sets. This reorganization is favored by multiple interactions as that of chromatin with HP1, BAF1, LBR, and with LEM-domain containing factors, as LAP2β. At this stage also starts the deposition of nuclear pore complex components in the reorganizing nuclear membrane (Figure 3(A)) [158–162].

Cytological and biochemical studies have shown that nuclear membranes are detected around chromosome sets by 5 minutes after the onset of anaphase, as established by the analysis of nuclear membrane-associated factors as emerin and LBR [163]. On the other hand, nuclear pore complex function is detected at about 8 minutes after anaphase onset [164]. Moreover, microscopy data have highlighted that, during late anaphase and successive telophase, two regions can be identified around the chromosome sets, namely the core and non-core [164]. The core can be further subdivided into inner and outer core with respect to the single chromosome set [162]. During late anaphase the core region is enriched of specific nuclear membrane-associated factors, namely BAF1, LAP2α, emerin and lamin A/C [159,161,163,165,166]. On the other hand, the non-core area contains nuclear pore complex components, lamin B and LBR (Figure 3(B)) [161,162,164,166]. While the non-core region is interlinked with the endoplasmic reticulum, the core region drives the organization of the ESCRT machinery and its functioning during telophase. Here, LEM2 is the driver for the recruitment of the ESCRT factor CHMP7 [161,164,167], which then controls the recruitment, in the final stages of mitosis, of the other components of the ESCRT machinery, to eventually contribute to the finalization of nuclear membrane sealing (Figure 3(A)) [168–171]. It was suggested that this process is governed by LEM2-dependent condensation of this factor on spindle microtubules [167,172].

Telophase closes the process of mitosis and prepares the daughter cells to their interphase. At this stage, a new phosphorylation status is required, and kinase activities are regulated accordingly. CDK1 activity, for example, decreases, which, in turn, drives the reduction of other kinases, which were upregulated in the earlier stages of mitosis [98,142,173,174]. In telophase, chromosomes progressively decompactify to return in the format of chromatin (Figure 3(B)). The modulation of condensin is critical to this process [175,176]. During telophase, the nuclear membrane reassembly becomes evident along with its association with the decondensing chromosomes. The spindle fibers are removed. This aspect in its latter stages depends on ESCRT activity [177]. Indeed, CHMP7 and the other ESCRT members CHMP2A, CHMP4C and CHMP8 (IST1) are recruited at the core and in turn recruit Spastin, the ATPase required to severe residual spindle microtubule fibers at the reforming nuclear membrane (Figure 3(B)) [169–171,178].

Interestingly, if the process of nuclear membrane reorganization and chromosome distribution are not correctly coordinated, for example, in the presence of a lagging chromosome, not only an independent micronucleus will form in the daughter cells but, also, the membrane of the micronucleus and that of the major nucleus will differ profoundly in their composition and mechano-biological properties [179–181]. This, as other studies, underline the importance of studying mitosis both in a vertical and in a horizontal manner, so to take into consideration the complexity of each sub machinery, along with the interlinks among the multiple complexes controlling the dynamic processes affecting DNA and organelles during mitosis.

Finally, while the nuclear membrane is progressively being reorganized, the tubulin composed midbody evolves into a progressively thinner structure between the daughter cells. Here the ESCRT machinery is also recruited, and the ESCRT subunits organize around the central area of the midbody as ring-like structures that eventually evolve into spirals. These spiralizing structures contribute to the final stage of cytokinesis, i.e. abscission, which ends with the liberation of two independent daughter cells (Figure 3(B)) [182–187].

## Dynamics of telomeres in early stages of mitotic division

While the mitotic distribution of chromosomes has been described in early times, the elements or events that control the post-mitotic reorganization of the genome are yet ongoing area of investigation, that involves many disciplines and levels of analysis. Particularly intriguing are the studies focusing on telomeres and centromeres. Indeed, these repetitive, specialized genomic portions are involved in driving the correct repartition and organization of the genome. However, the dissection of telomere dynamics along with its impact on the post-mitotic genome is still incomplete especially when considering mammalian mitosis.

**Figure 4.**
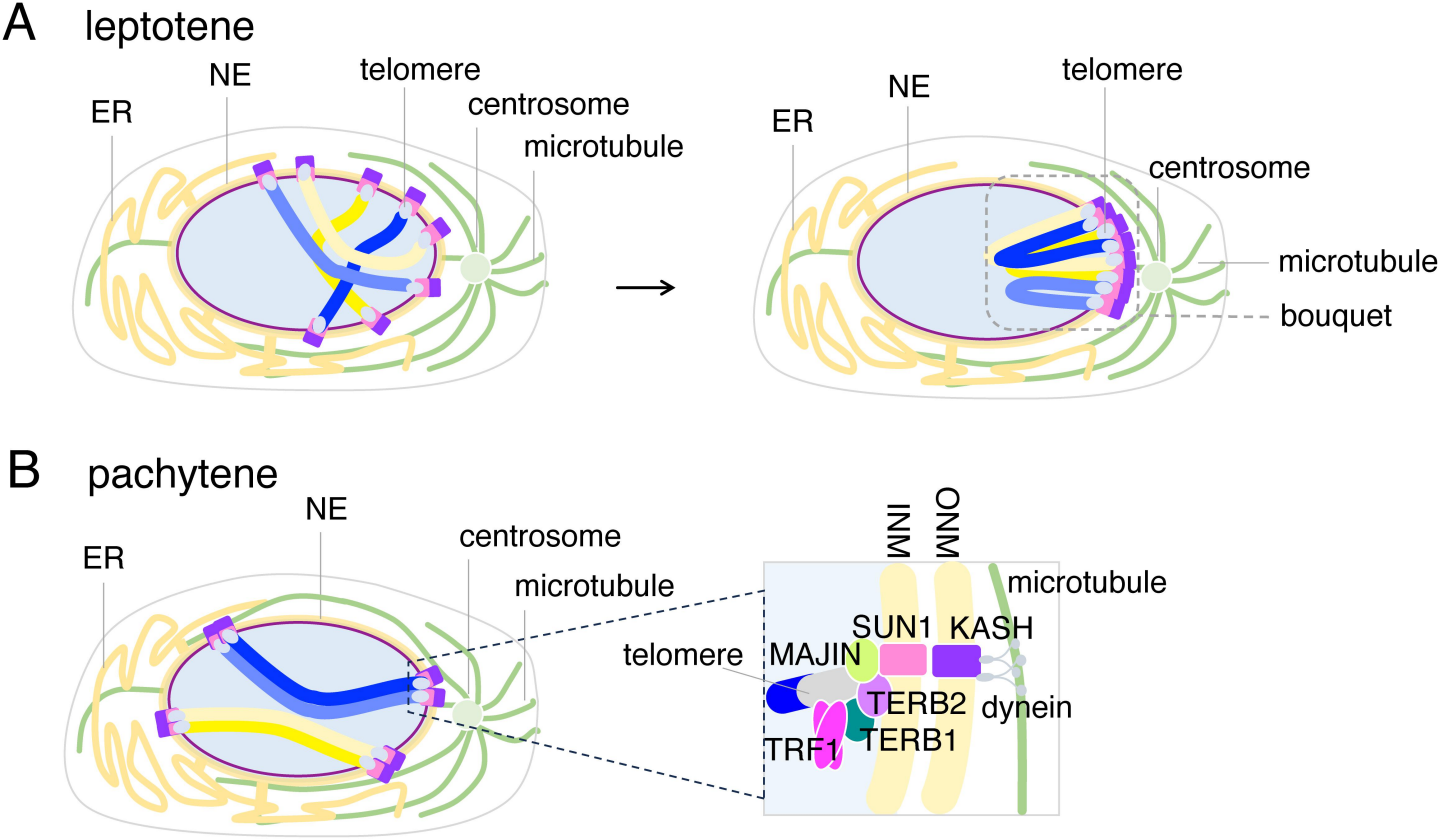
Telomere dynamics during meiotic bouquet formation. (A-B) Schematic representation of telomere organization during mammalian meiotic stages, leptotene (A) and pachytene (B). Telomeres establish interactions with the nuclear membrane during interphase, then telomeres congress at one pole of the nuclear membrane in meiosis prophase (leptotene) to form the bouquet. This structure facilitates chromosome movements. During pachytene, telomeres remain anchored at the NE while homologous chromosomes are fully paired. Telomere attachment to the nuclear envelope is mediated by the interaction between telomeric proteins (TRF1) and nuclear envelope components, including SUN and KASH domain proteins. During meiotic progression, the shelterin complex is replaced by the TERB1/2-MAJIN complex (B, inset). ER, endoplasmic reticulum; NE, nuclear envelope; ONM, outer nuclear membrane; INM, inner nuclear membrane.

On the other hand, the dynamics of chromosomes and telomeres in meiosis has been described and dissected both in model organisms and in humans. Indeed, the first data were shown at the beginning of last century when a peculiar structure, then referred as the chromosomal bouquet, was described in flatworm meiosis [188]. This structure is conserved in yeast, plants, fish and humans. To form this structure, telomeres establish interactions with the nuclear membrane during interphase, then telomeres congress at one pole of the nuclear membrane in meiosis prophase (Figure 4(A)) [189–193]. The interpretation of the function of the bouquet is an evolving area of study. The general concept, based on the fact that the formation of the bouquet coincides with the chromosome pairing stage, has suggested the hypothesis that this peculiar and temporally defined telomeric organization is required to contribute to fulfill the mechanisms ensuring homologous recombination [191]. However, subsequent studies, based on the analysis of genetic mutants of Saccharomyces pombe, extended the interpretation of the role of the bouquet. The genetic work on pombe in fact showed that the bouquet controls also the spindle apparatus via the spindle microtubule organizing center [190,194]. Studies in yeast have also identified a mechanistic relation between centromere positioning and telomere bouquet formation, that, in turn, impacts on meiotic reprogramming [195]. Furthermore, in fission yeast, centromeres interact with the nuclear envelope, regulate nuclear envelope disassembly and spindle pole body positioning, suggesting some parallel elements with telomeres [196]. A reference study, that discusses extensively the telomere and centromere behavior in mitosis and meiosis in yeast, is the review by Hou and Cooper. The present review adds updates on the one side on telomere–nuclear envelope–ESCRT-related mechanisms, and, on the other side, it extends to the analysis of mammalian systems [197].

In fact, as in yeast, during mammalian gametogenesis telomeric bouquet structures can be identified. At the molecular level, telomeric factors have been directly involved in the process. Namely, for example, the germ cell-specific knockout of the telomeric factor TRF1, drives recombination defects and meiotic division arrest. Indeed, multiple factors on the one side mediate the enrichment of telomeres at the nuclear periphery in interphase and contribute to the meiotic bouquet subsequently [198,199]. These factors include not only intranuclear elements as TRF1, but also nuclear membrane traversing factors, which create a link between the genome and the outer cytoskeleton as the SUN and KASH domain proteins (Figure 4(B)) [193,200–202]. Moreover, in mice, with the progression of mammalian meiosis, the germ cell shelterin complex is progressively substituted by the multi-subunit TERB1/2-MAJIN complex in a process named telomere cap-exchange. MAJIN is a driver of meiotic tethering of telomeres to the nuclear membrane and of the successive stages of their meiotic distribution (Figure 4(B)) [193,203,204].

**Figure 5.**
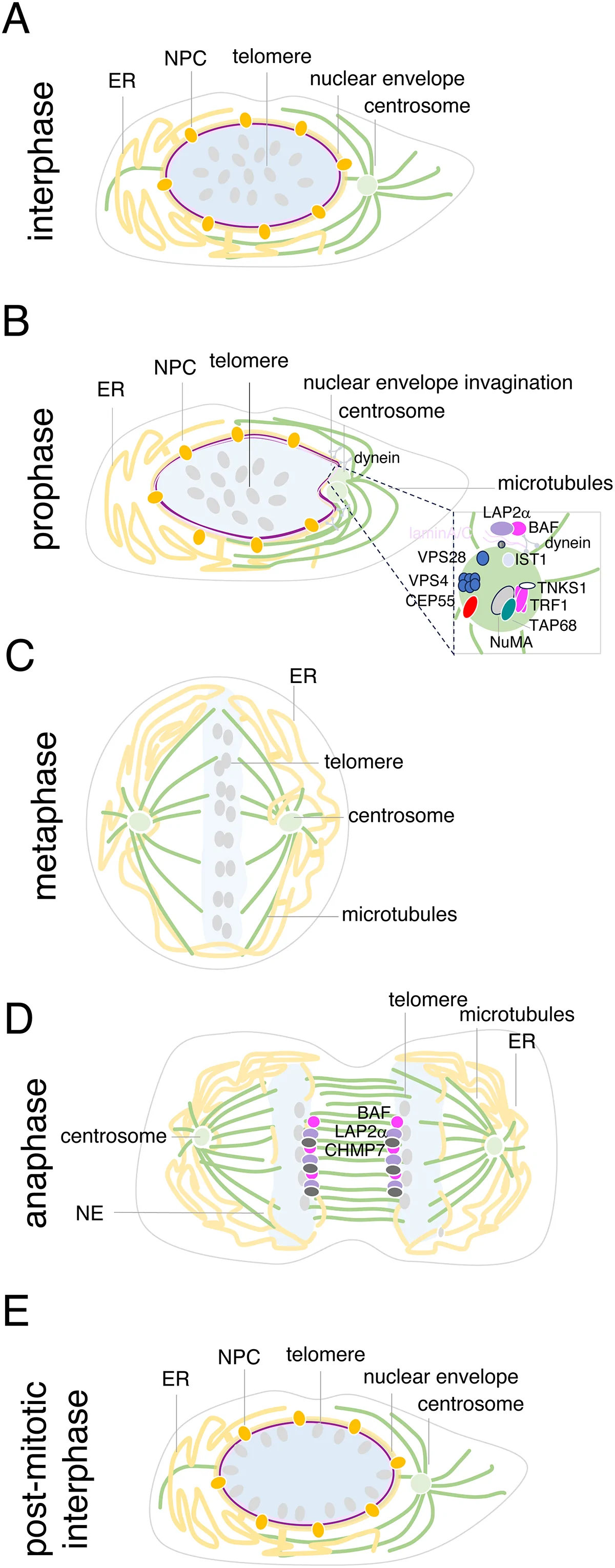
Dynamics of telomeres during mitotic division. In human cells, telomere localization dynamically changes across the cell cycle. During interphase (A), telomeres are distributed within the nucleoplasm, often associated with the nuclear matrix. During prophase and metaphase (B-C), telomeres align at the metaphasic plate, with TRF1 levels increasing and localizing also to the mitotic spindle and centrosomes (B, inset) During anaphase (D), telomeres are found at sites of nuclear envelope reformation, where they co-localize with LAP2α, BAF1 and CHMP7. During early post-mitotic stages (E), telomeres remain closely associated with the reforming nuclear envelope (<500 nm), highlighting a coordinated link between telomere positioning and nuclear envelope reassembly.

The prototypical characteristics of the telomeric meiotic bouquet inspired the search for parallel structures in mitotic cells. Even though, neither a canonical bouquet nor a similar structure has yet been described in mitotic cells. However, some data in mitosis reflect the connection between telomere dynamics and microtubules described for meiotic prophase. In Saccharomyces pombe mitosis, which is closed in this model organism, the phosphorylation of the telomeric factor Rap1 induces telomere dissociation of nuclear membrane tethered telomeres and contributes to the correct distribution of the chromosomes in the daughter cells (Table 1) [216]. In mammalian mitosis, components of the telomeric apparatus have been shown to localize at spindle poles/centrosomes, namely the shelterin TRF1, the telomeric accessory factor TNKS1 and the co-factor TAP68 (Table 1) [209–211]. TNKS1 detection at spindle poles was associated with PARPsylation and interaction with NuMA, a factor playing a critical role in spindle assembly (Figure 5) [233–237]. Moreover, a link was established in mammalian cells between the shelterin Rap1 and the protein SUN1 (Table 1) [207] This aspect is paralleled in Caenorhabditis elegans where it has been shown that POT-1 anchors telomeres to the nuclear periphery through SUN-1, illustrating that LINC-mediated chromosome–nuclear envelope interactions are not restricted to canonical meiotic bouquet systems [213]. Together, these data suggest a possible early contribution of telomeres and associated factors in the early control of chromosome segregation.

**Table 1.** Insights into telomere positioning throughout the cell cycle in somatic cells of different model systems.

Cell cycle stage	Telomere position	Telomeric factor involved	Other factors involved	Organism	Cellular system	Techniques	Type of evidence	Ref
Interphase	Nucleoplasm			*M. musculus*	Lymphocyte	FISH on FACS sorted cells	Localization	[205]
Prophase	Bimodal distribution: some cluster at the center some are peripheral			*M. musculus*	Lymphocyte	FISH on FACS sorted cells	Localization	[205]
Interphase	Nucleoplasm in association with nuclear matrix	TRF	Nuclear matrix	*H. sapiens*	HeLa	FISH; Biochemical assays; IF-FISH	Localization; biochemistry	[206]
Lateanaphase-early G1	Reforming nuclear envelope (distances < 500 nm)	Rap1	SUN1	*H. sapiens*	IMR-90 and HeLa	Live cell imaging tagged TRF1; Co-IF TRF1-Lamin A/C; FISH-IF; modified telomeric ChIP; Co-IP tagged Rap1-Sun1	Localization; biochemistry	[207]
Interphase G2	Away from nuclear envelope			*H. sapiens*	HeLa	3D SIM and 3D modelling	Localization	[208]
Early anaphase	Reforming nuclear envelope		BAF-Lap2a	*H. sapiens*	HeLa, NRK	Co-IF TRF2-Lap2a; Co-IF Lap2a-BAF; Co-IP Lap2a-BAF	Localization; biochemistry	[161]
Pro-metaphasemetaphase	metaphasic plate	TRF1	TNKS1	*H. sapiens*	HeLa	FISH	Localization	[41] [42]
Metaphase		TRF1 level increases and it is located at mitotic spindle and at centrosomes	Microtubules EBP1; Mad1 and Nek2 TAP68;	*H. sapiens;* *M. musculus*	HeLa	Co-IF TRF1-tubulin; in vitro enzymatic tests; IP and GST-pull down Co-IP and GST pull down TRF1-EBP1; yeast-two hybrid;in vitro biochemical assay;Co-IF TRF1-TAP68; Co-IP TRF1-TAP68	Localization; biochemistry	[209,210] [211,212]
Interphase	Nuclear envelope	POT1	SUN1; GEI-17	*C. elegans*	Embryo; differentiated cells	FISH; IF-FISH; FISH in mutant strains	Localization; localization on perturbed strains	[213]
Interphase	Nuclear envelope	Taz1-Rap1	Bqt4-Bqt3; Man1, Lem2	*S. pombe*		Yeast two-hybrid assay;IF;mutant strains;FISH	Localization; biochemistry; functional analysis of perturbed strains	[214,215]
Mitosis	Away from nuclear envelope	Cdc2 dependent P of Rap1	Bqt4-Bqt3	*S. pombe*		Biochemical analysis; IF	Localization; biochemistry	[216]
Interphase	Nuclear envelope	yKu70-Ku80; Sir3 and Sir4	Mps3; Tlc1; Est1; Est2 (S-phase) Esc1p; SUMO E3 ligase Siz2	*S. cerevisiae*		Live cell imaging; FISH; mutant strains	Localization; biochemistry; functional analysis of perturbed strains	[217–224]
From late S-phase to the end of mitosis	Away from nuclear envelope	yKu70-Ku80;	DNA replication factors	*S. cerevisiae*		Live cell imaging; mutants strains	Localization; localization on perturbed strains	[225]
Interphase	Nuclear envelope	TEBP α-TEBP β	SNS I-III	*S. lemnae* (ciliate)		In vivo and in vitro; RNAi experiments	Functional analysis of perturbed strains	[226–228]
From late S-phase to the end of mitosis	Away from nuclear envelope	TEBP α-TEBP β	SNS I-III	*S. lemnae* (ciliate)		Biochemical studies	Biochemistry	[226–228]
Ana-telophase	Spindle pole		microtubules	*T. brucei*		FISH	Localization	[229]
Interphase	Cluster at NE		NUP1 - NUP2	*T. brucei*		FISH; IF-FISH; RNAi	localization; localization on perturbed strains	[229–231]
Interphase	Cluster at NE at a pole of the nucleus			*P. sativum* *V. faba*		FISH	Localization	[232]

## Dynamics of telomeres in late stages of mitotic division

Going back to early studies, it was reported that chromosomes of *Allium cepa* display a sequential migration at opposite poles, starting with the central regions and ending with telomeric regions [238]. In Tetrahymena thermophila, a seminal study showed that telomeres are essential for chromosome separation in anaphase. The study exploited telomerase mutants to prove that aberrant telomere metabolism generates a delay or block of the anaphase stage in mitosis (Table 1) [239]. More recent experiments focused on the study of shelterin implication in the anaphase stage. Indeed, for example, aberrant expression of the shelterin TRF1 causes aberrant separation of telomeres in anaphase, resulting in anaphase DNA bridges [240]. TNKS1 was also implicated in anaphase defects. Indeed, the reduction of TNKS1 causes persistent telomere cohesion in anaphase, and delay in anaphase progression [42,44,241]. An interesting study in yeast further demonstrated an interplay between condensin and Taz1, the yeast orthologue of the human shelterin TRF1. The study showed that Taz1 controls the concentration of condensin at telomeres, affecting sister-telomere separation in anaphase (Table 1) [242]. One further study on telomeres in mitosis correlates telomere protecting factors with prolonged mitotic arrest [243].

Parallel to cytogenetic studies on chromosome distribution and anaphase defects, are those focusing on telomere dynamics in the frame of nuclear membrane disassembly and reassembly occurring during mitosis (Figure 5(A–E)). Indeed, even though the dynamics of telomeres in mammalian cells has been difficult to define, some studies challenged this subject. It was demonstrated that, in anaphase and, successively, in telophase, when the inner membrane proteins are reorganizing into a nuclear enveloping structure, LAP2α and BAF1 enrich as foci at chromosome ends (Table 1) [161,163,164,244]. Further, more recent studies based on proximity labeling screenings, confirmed that LAP proteins and BAF1 interact with telomeres in anaphase (Table 1) [208]. Importantly, BAF1 also intercepts biochemically and dynamically the assembly of the ESCRT factors during nuclear membrane assembly. Indeed, the ESCRT CHMP7, and subsequently CHMP4B and CHMP2A, depend on the anaphase enrichment of BAF1 and LEM2 at the core of chromatin discs (Figure 5(D)) [167–170]. Consistently with the interdependence of these factors with telomeres, the reduced expression of CHMP7 drives telomeric aberrations [245].

Finally, some studies establish an association of telomeres with the nuclear periphery of interphase somatic cells. Early evidence anticipated this concept by identifying an association of telomeres with the nuclear matrix (Table 1) [206,246,247]. Successively, cytological and proximity labeling studies confirmed an enrichment of telomeres at the nuclear rim early post mitotic stage (Figure 5(E) and Table 1) [207]. A recent preprint [248] proposes that telomeres act as initiation sites for the reassembly of nuclear envelope, and that depletion of TRF1 or TRF2 alters the spatial pattern of nuclear envelope reassembly and increases nuclear alterations. This work, although in its preliminary format, fits particularly well the concepts discussed in this review and points to a possible explanation on how telomere mislocalization during mitotic division leads to nuclear alterations putatively creating a vicious cycle generating telomere aberrations.

## Concluding remarks

Since a critical role of telomeres structure, of their length and organization has been described in association with physiological aging, studies on telomere dynamics are expected to impact also on the design of strategies aiming at challenging and reversing aging. Moreover, telomere studies can be instrumental also to dissect some premature aging syndromes. In fact, for example, mutations in lamin genes drive telomeric alterations and premature aging syndromes [249,250]. The most paradigmatic case is that of the Hutchinson Gilford Progeria, a rare syndrome due to a dominant mutation of the laminA/C encoding gene [251,252]. The progeroid, prematurely aging cells exhibit profoundly altered nuclei, which, consistently with the interplay between the nuclear membrane and the organization of chromatin, also display chromatin defects, including telomeric aberrations [253,254]. Other mutations, either in the LMNA gene or in other nuclear membrane-associated genes, as mutations in ZMPSTE24 can also induce nuclear membrane defects and consequent chromatin disorganization, generating diseases including restrictive dermopathy, mandibuloacral dysplasia type A and B, and the atypical Werner syndrome [255]. Another progeroid disease that is particularly interesting, when considering the dynamics of the mitotic elements analyzed in this review, is the Nestor-Guillermo progeria syndrome. In fact, this type of syndrome is determined by a mutation of the BAF1 encoding gene [256,257]. It can be cautiously hypothesized that telomere–nuclear envelope interface, BAF1 restoration pathways, or ESCRT-related mechanisms could represent conceptual entry points for therapeutic intervention in such diseases.

In conclusion, although the positioning and dynamics of telomeres is clarified in meiosis, in mammalian mitotic cells the positioning and the dynamics of telomeres remain partially elusive. This notwithstanding, it has been demonstrated that the distribution of telomeres is non-randomic, and genetically and physically interlinked with multiple factors, which intercept microtubules and elements of the nuclear membrane. More specifically, different experimental levels, including (i) telomere proximity to the reforming nuclear envelope, (ii) biochemical or proximity-labeling evidence, (iii) functional perturbation studies affecting telomere integrity or nuclear envelope reassembly and (iv) direct causal evidence showing that telomeres spatially instruct nuclear envelope sealing or ESCRT recruitment, suggest a role for telomeres in orchestrating some determinant aspects of mitosis. Altogether, because of its yet incomplete understanding and, at the same time, because of the central role of telomeres in the onset of the premature or naturally aging cell, the full comprehension of their dynamics in human mitosis and of the factors controlling this aspect deserves interest, further studies and technological efforts.

## Data Availability

Data sharing is not applicable to this article as no new data were created or analyzed in this study.
